# Whole-body low-dose CT recognizes two distinct patterns of lytic lesions in multiple myeloma patients with different disease metabolism at PET/MRI

**DOI:** 10.1007/s00277-018-3555-7

**Published:** 2018-12-11

**Authors:** Renato Zambello, Filippo Crimì, Albana Lico, Gregorio Barilà, Antonio Branca, Annamaria Guolo, Cristiano Varin, Roberto Vezzaro, Lucia Checuz, Vanna Scapin, Tamara Berno, Marco Pizzi, Alberto Ponzoni, Ercole De Biasi, Stefania Vio, Gianpietro Semenzato, Pietro Zucchetta, Carmelo Lacognata

**Affiliations:** 1 Department of Medicine, Hematology Section and Clinical Immunology Branch, Padua University School of Medicine, Via Giustiniani 2, 35128 Padova, Italy; 2 Department of Medicine, Institute of Radiology, Padua University School of Medicine, Padua, Italy; 3 Department of Statistical Sciences, Padua University School of Medicine, Padua, Italy; 4 Department of Environmental Sciences, Informatics and Statistics, Ca’ Foscari University, Venice, Italy; 5 Department of Medicine, Surgical Pathology and Cytopatology Unit, Padua University School of Medicine, Padua, Italy; 6 Department of Medicine, Hematology Section, Camposampiero Hospital, Padua, Italy; 7 Department of Nuclear Medicine, Padua University School of Medicine, Padua, Italy

**Keywords:** Multiple myeloma, Lyltic bone lesion, WB-LDCT, PET/MRI

## Abstract

We evaluated differences in density and ^18^F-FDG PET/MRI features of lytic bone lesions (LBLs) identified by whole-body low-dose CT (WB-LDCT) in patients affected by newly diagnosed multiple myeloma (MM). In 18 MM patients, 135 unequivocal LBLs identified by WB-LDCT were characterized for inner density (negative or positive Hounsfield unit (HU)), where negative density (HU < 0) characterizes normal yellow marrow whereas positive HU correlates with tissue-like infiltrative pattern. The same LBLs were analyzed by ^18^F-FDG PET/DWI-MRI, registering DWI signal with ADC and SUV max values. According to HU, 35 lesions had a negative density (− 56.94 ± 31.87 HU) while 100 lesions presented positive density (44.87 ± 23.89 HU). In seven patients, only positive HU LBLs were demonstrated whereas in eight patients, both positive and negative HU LBLs were detected. Intriguingly, in three patients (16%), only negative HU LBLs were shown. At ^18^F-FDG PET/DWI-MRI analysis, negative HU LBLs presented low ADC values (360.69 ± 154.38 × 10^−6^ mm^2^/s) and low SUV max values (1.69 ± 0.56), consistent with fatty marrow, whereas positive HU LBLs showed an infiltrative pattern, characterized by higher ADC (mean 868.46 ± 207.67 × 10^−6^ mm^2^/s) and SUV max (mean 5.04 ± 1.94) values. Surprisingly, histology of negative HU LBLs documented infiltration by neoplastic plasma cells scattered among adipocytes. In conclusion, two different patterns of LBLs were detected by WB-LDCT in MM patients. Both types of lesions were indicative for active disease, although only positive HU LBL were captured by ^18^F-FDG PET/DWI-MRI imaging, indicating that WB-LDCT adds specific information.

**Electronic supplementary material:**

The online version of this article (10.1007/s00277-018-3555-7) contains supplementary material, which is available to authorized users.

## Introduction

In multiple myeloma (MM) patients, the detection of lytic bone lesions (LBLs) represents a criterion to define symptomatic MM requiring treatment, even in the absence of other clinical symptoms [1–3]. In the workup of MM patients, whole-body planar X-ray has been long considered the gold standard for detecting bone involvement. More recently, whole-body low-dose computerized tomography (WB-LDCT) has been used with increased frequency in patients with plasma cell dyscrasias, in order to detect the presence of bone disease, including punched out LBLs, diffuse osteopenia, fractures and, rarely, osteosclerosis, also revealing the presence and size of myelomatous soft tissue masses. The sensitivity of WB-LDCT has been proven to be higher than that of planar X-ray, and it has been suggested that this technique might actually represent the gold standard for evaluation of LBLs [4, 5].

WB-LDCT is a “morphological technique” with a high sensitivity for detection of even small osteolytic lesions and their characterization in terms of number, dimension and internal density. Most authors consider as myelomatous lesions those osteolytic lesions that have a positive tissue-like radiodensity, expressed as Houdsfield unit (HU) due to plasma cells infiltration [6]. It is well known that most intramedullary space in the appendicular and axial skeleton is normally replaced by fatty marrow in healthy adults [7]. By CT imaging evaluation, this feature results in lower CT attenuation values (CTav) than the density of water (which corresponds to HU 0). In contrast, when this space is infiltrated by neoplastic cells as in MM patients, as a consequence of the destruction of mineralized bone, the marrow lesion densities are characterized by a positive value (HU > 0), consistent with the presence of solid (myelomatous) tissue [8]. Interestingly, Nishida et al. [8] recently demonstrated that medullary abnormalities of appendicular skeleton with HU > 0 were correlated with tumour cell burden of myeloma assessed by laboratory parameters, namely high-risk cytogenetic and Durie–Salmon (DS) III, providing a relationship between these values in patients with MGUS and MM [8]. In literature, IMWG criteria do not specify whether density of lytic lesions should be measured and merely state to define only morphologically this kind of lesions as typical pounched-out osteolytic areas with diameter > 5 mm without reactive sclerosis of the surrounding bone [9]. In particular, the clinical significance of > 5 mm lytic lesions with negative (HU < 0) density has never been addressed. As a matter of fact, we were impressed by the number of lesions with HU < 0 that could be demonstrated in some patients with otherwise active myeloma, sometimes associated with the more typical HU > 0 lesions. To better define the specific contribution to WB-LDCT to skeletal survey in a series of newly diagnosed MM patients, we retrospectively evaluated the features of LBLs detected by WB-LDCT and compared the pattern with ^18^F-FDG PET/DWI-MRI, which fuse together two very highly sensitive functional techniques suggested by IMWG to assess bone involvement in MM patients [10, 11].

## Methods

### Patients

We retrospectively selected 18 patients (12 males and 6 females) with mean age 57 years (range 42–73 years) affected by newly diagnosed MM (NDMM) who underwent both WB-LDCT and 18F-FDG PET/MRI. Bone marrow biopsy and laboratory tests were performed in all patients, and MM diagnosis was confirmed according IMWG criteria^8^. According to the Durie–Salmon staging system, 13 patients (72%) were classified as stage III. The characteristics of the included patients are reported in Table 1. No patient received bisphosphonates, and chemo or radiotherapy before WB-LDCT and PET/MRI were performed. Furthermore, any patient presented symptoms or signs of infection or inflammation, uncontrolled hyperglycemia, or received steroid within few days before the exams. The mean time between the two examinations was 10.7 ± 9.1 days, range 0–27 days. In two patients for whom an initial policy of wait and see was adopted (cases 17 and 18), two different evaluations were available before starting therapy. Approval for retrospective evaluation and publication was obtained from our institutional research ethics board. The written informed consent requirement was waived. This study followed the Declaration of Helsinki, and all patients gave written informed consent.

**Table 1:** Clinical features of patient’s cohort

Patients	Clinical features
Mean age	57 years (42–73 years)
Sex	12 males, 6 females
Isotype MM	11 patients, IgG/κ
	3 patients, IgA/κ
	1 patient, IgG/λ
	1 patient, IgA/λ
	1 patient, IgD/κ
	1 patient, IgD/λ
SLiMCRAB criteria	
PCs > 60%	13 patients (72%)
Ratio κ/λ > 100	7 patients (39%)
MRI focal lesion >1	15 patients (83%)
PET positivity	13 patients (72%)
Hypercalcemia	2 patients (11%)
Renal failure	1 patient (5%)
Anemia	9 patients (50%)
Bone disease (osteolysis)	18 patients
ISS	I, 9 patients
	II, 6 patients
	III, 3 patients
Durie–Salmon	IA, 2 patients
	IIA, 3 patients
	IIIA, 12 patients
	IIIB, 1 patient
Cytogenetic	Standard risk, 14 patients
	High risk*, 4 patients

*High cytogenetic risk was defined by the presence of del17p, *t*(4; 14), *t*(14; 16) or gain1q

### WB-LDCT and ^18^F-FDG PET/DWI-MRI acquisition protocol

The WB-LDCT examinations were performed with a 128 Slice CT scanner (Siemens Somatom Definition) while ^18^F-FDG PET/DWI-MRI studies were performed with an integrated ^18^F-FDG PET/DWI-MRI scanner (Siemens Biograph mMR), which allows simultaneous acquisition of ^18^F-FDG and DWI-MRI data.

Acquisition protocol for WB-LDCT was as follows: X-ray tube voltage 120 kV, X-ray tube current 35 mAs, collimation 16 × 1.5 mm, pitch = 1, and slice thickness 2 mm. In patients with body weight above 80 kg, the X-ray tube current was increased to 50 mAs. The scan length extended from hands to feet. The mean scan duration was 50 s. Both smooth (B30f) and sharp (B70f) kernels were used for reconstructions. Both smooth and sharp kernel images underwent also multiplanar reconstructions (MPRs) in coronal and sagittal planes.

MRI imaging protocol was the following: whole-body axial T2-weighted half-Fourier acquisition single-shot turbo spin-echo (HASTE) sequence, sagittal T1-weighted spin-echo sequences, axial T1-weighted spoiled gradient recalled sequence with fat signal suppression (VIBE), coronal turbo inversion recovery magnitude (TIRM) sequence, and axial spin-echo single-shot echo-planar imaging sequences with diffusion-sensitizing gradients (DWI) applied along *x*, *y*, and *z* axes (*b* value 50 and 1000 s/mm^2^).

Before PET imaging acquisition, patients were instructed to fast for at least 6 h before the injection of 3 Mbq/kg of ^18^F-FDG, after blood-glucose levels being checked (maximum 200 mg/dl). The scanning began 60 min after ^18^F-FDG injection, and images were acquired from feet to vertex, according to published protocol [12].

### Image analysis

Two expert radiologists examined every WB-LDCT identifying every lytic bone lesion > 5 mm and characterized it for dimensions and internal densitometry (negative or positive HU). One radiologist and one nuclear medicine physician evaluated together the same lesions with ^18^F-FDG PET/DWI-MRI images registering T1w signal, TIRM signal, DWI signal, mean apparent diffusion coefficient (ADC) value, and standardized uptake volume (SUV) max value. Concerning MRI, ADC value was considered pathological when higher than 600 × 10^−6^ mm^2^/s, as previously reported [13, 14]. Considering PET, a SUV max value > 2 within osteolytic CT areas higher than 5 mm was considered as indicative of PET positivity [15].

### Histological evaluation

In all cases, bone marrow biopsy evaluation was performed as a part of the staging workup for MM at iliac spine. In selected patients (cases 16, 17, and 18), typical HU < 0 LBLs underwent to needle biopsy. In detail, 3-μm-thick slides were obtained from formalin-fixed, decalcified paraffin-embedded marrow samples. Following the routine protocol for bone marrow examination, the slices were stained with H&E, PAS, and Giemsa. Marrow fibrosis was assessed by reticulin stain. The immunohistochemical characterization of plasma cells was performed using the following antibodies: anti-CD138 (clone MI15 Dako, Glostrup, Denmark), anti-MUM1 (clone MUM1p, Dako, Glostrup, Denmark), anti-CD56 (clone 504 Leica, Milan, Italy), anti-cyclin D1 (clone EP12, Dako, Glostrup, Denmark), anti-CD20 (clone L26, Dako, Glostrup, Denmark), anti-CD79a (clone 11E3, Leica, Milan, Italy), anti-CD3 (clone LN10, Novocastra, Milan, Italy), and Mib1 (clone Mib1 Dako, Glostrup, Denmark). An anti-CD68 antibody (clone PGM1, Dako, Glostrup, Denmark) was used to assess the presence and distribution of peri-trabecular osteoclasts.

### Statistical analysis

Statistical analyses were performed with the R software (R Core Team. “R: A language and environment for statistical computing.” R Foundation for Statistical Computing, Vienna, Austria. 2018. URL https://www.R-project.org/). Statistical analysis based on linear regression with robust standard errors accounting for dependence between observations from the same patient was performed. A *p* value < 0.05 was accepted as significant.

## Results

### Clinical features of patients

Together with a positive WB-LDCT showing the presence of lytic lesions, 16 out of 18 patients (89%) presented additional criteria for active MM [9], i.e., a positive MRI exam (83%), marrow plasma cell infiltration > 60% (72%), and anemia (50%) (Table 1). All but two patients were further treated according to a first-line regimen including a PI inhibitor as induction therapy. Two cases, initially classified as high-risk smoldering MM, started therapy following 1-year follow-up.

### Analysis of lytic bone lesions

One-hundred thirty-five LBLs with > 5 mm main diameter were recognized by WB-LDCT. Thirty-five lesions (mean size 11.09 mm ± 4.45) had a negative density (mean − 56.94 HU; SD ± 31.87 HU) while 100 lesions (mean size 17.36 mm ± 13.24) presented positive density (mean 44.87 HU; SD ± 23.89). Negative HU LBLs presented significant lower sizes towards positive HU LBLs (*p* < 0.01). The skeletal distribution of LBLs, distinguished according to WB-LDCT density (negative or positive HU), is reported in Table 2. Lesions with positive HU showed a trend toward a prevalent spine distribution as compared to those with negative HU (60% vs 38%, respectively (*p* < 0.05), whereas lesions with negative HU were more represented at the site of pelvis, although not significantly (45% vs 26%, *p* = 0.067).

**Table 2:** Distribution of lytic bone lesions (LBLs) in the skeleton

Location	LBL with HU > 0 (*n*)	LBL with HU < 0 (*n*)
Skull	2	0
Spine	*60* (*60%*)	*14* (*40%*)
Cervical spine	11	2
Dorsal spine	28	4
Lumbar spine	16	8
Sacrum	5	0
Pelvis	*26* (*26%*)	*16* (*45.7%*)
Long bones	2	0
Ribs	9	4 (11%)
Sternum	2	1 (3%)

Pelvis and Spine lesions were the most frequent sites of lytic lesions and signed in italics

As far as the LBL density, 8 patients (44.4%) had a mixed pattern with simultaneous coexistence of positive and negative lesions densitometries, 7 patients (38.9%) presented only positive LBL densitometries, and 3 patients (16.6%) presented only negative LBL densitometries (Table 3). Within the group with mixed pattern at WB-LDCT, 3 patients in addition presented extramedullary plasmocytoma whereas one patient rapidly evolved in plasma cell leukemia.

**Table 3:** Distribution of lytic bone lesions (LBLs) in each patient according to their HU value

Patients		HU > 0 LBL (*n*)	HU < 0 LBL (*n*)
Patients with coexistence of both HU > 0 and HU < 0 LBL	Patient 1	2	2
	Patient 2	12	1
	Patient 3	13	1
	Patient 4	10	2
	Patient 5	1	2
	Patient 6	3	7
	Patient 7	2	8
	Patient 8	1	4
Patients with only HU > 0 LBL	Patient 9	1	
	Patient 10	11	
	Patient 11	18	
	Patient 12	1	
	Patient 13	1	
	Patient 14	8	
	Patient 15	16	
Patients with only HU < 0 LBL	Patient 16		1
	Patient 17		1
	Patient 18		6

**Fig. 1:**
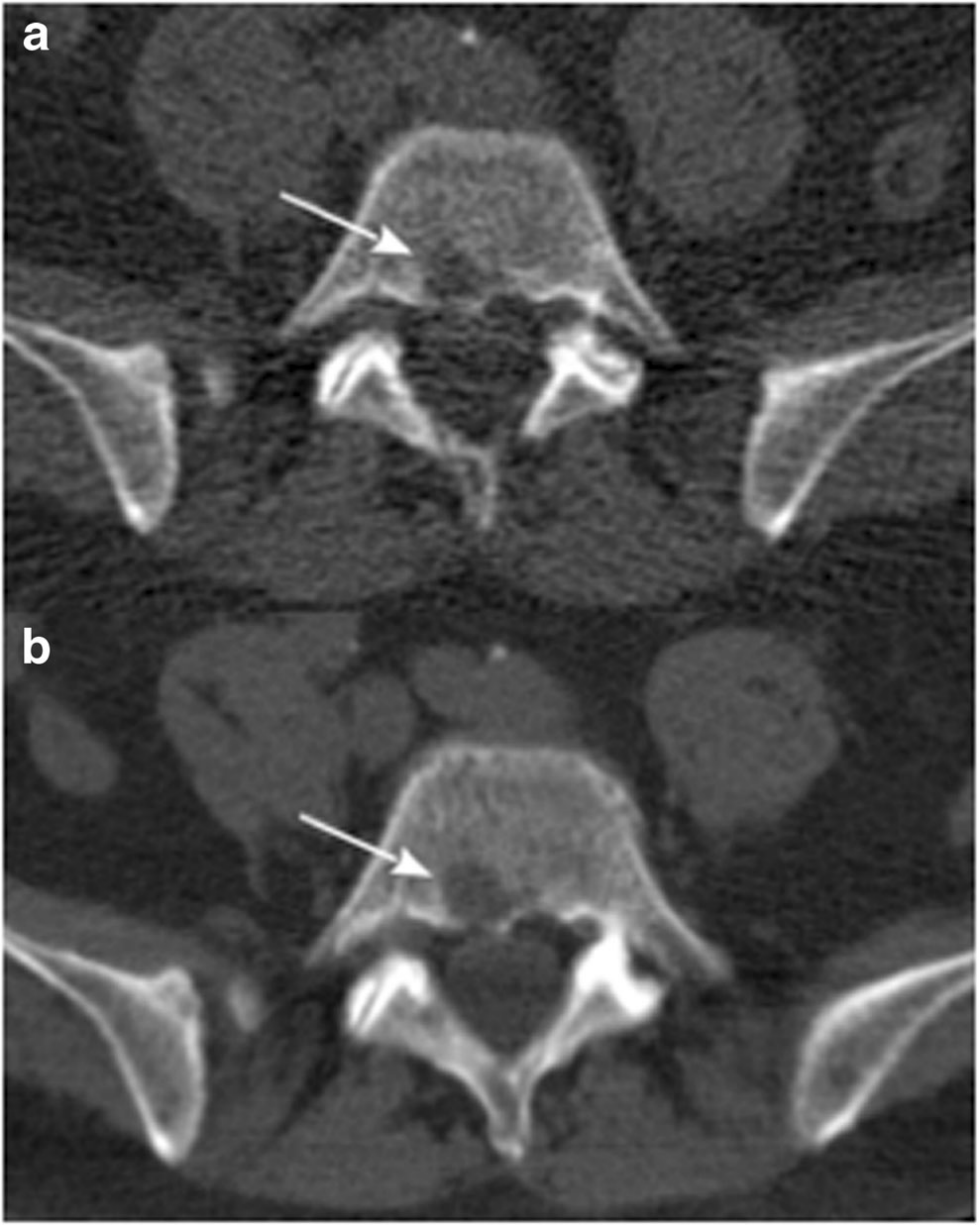
**a** WB-LDCT showing lytic bone lesion (white arrow) at L5 with negative densitometry of − 47.7 HU. **b** Ten months later the same lesion showing positive densitometry of 29.5 HU

Concerning patients with only negative densitometry (HU < 0) LBLs, the first patient (N°16) with one osteolytic lesion at iliac crest bone presented total PCs infiltration of bone marrow and anemia calling for starting therapy. The second patient (N°17) with only one osteolysis at L5 was otherwise classified as high-risk smoldering MM. At 10-month follow-up, the same CT lesion showed positive densitometry over other bone lesions (Fig. 1). This finding was associated with total PC bone marrow infiltration and anemia requiring starting therapy. The third patient (N°18) with six osteolytic lesions of spine had also 50% of malignant PCs in bone marrow. Due to PET and MRI negativity, this pattern of osteolytic lesions was initially considered of uncertain clinical significance, and patient started close monitoring. Ten months later, in presence of a biochemical progression, a second WB-LDCT showed an increased in number and size of previously negative densitometries LBL and the development of other areas with positive densitometry.

### Correlation between WB-LDCT and PET/MRI

**Fig. 2:**
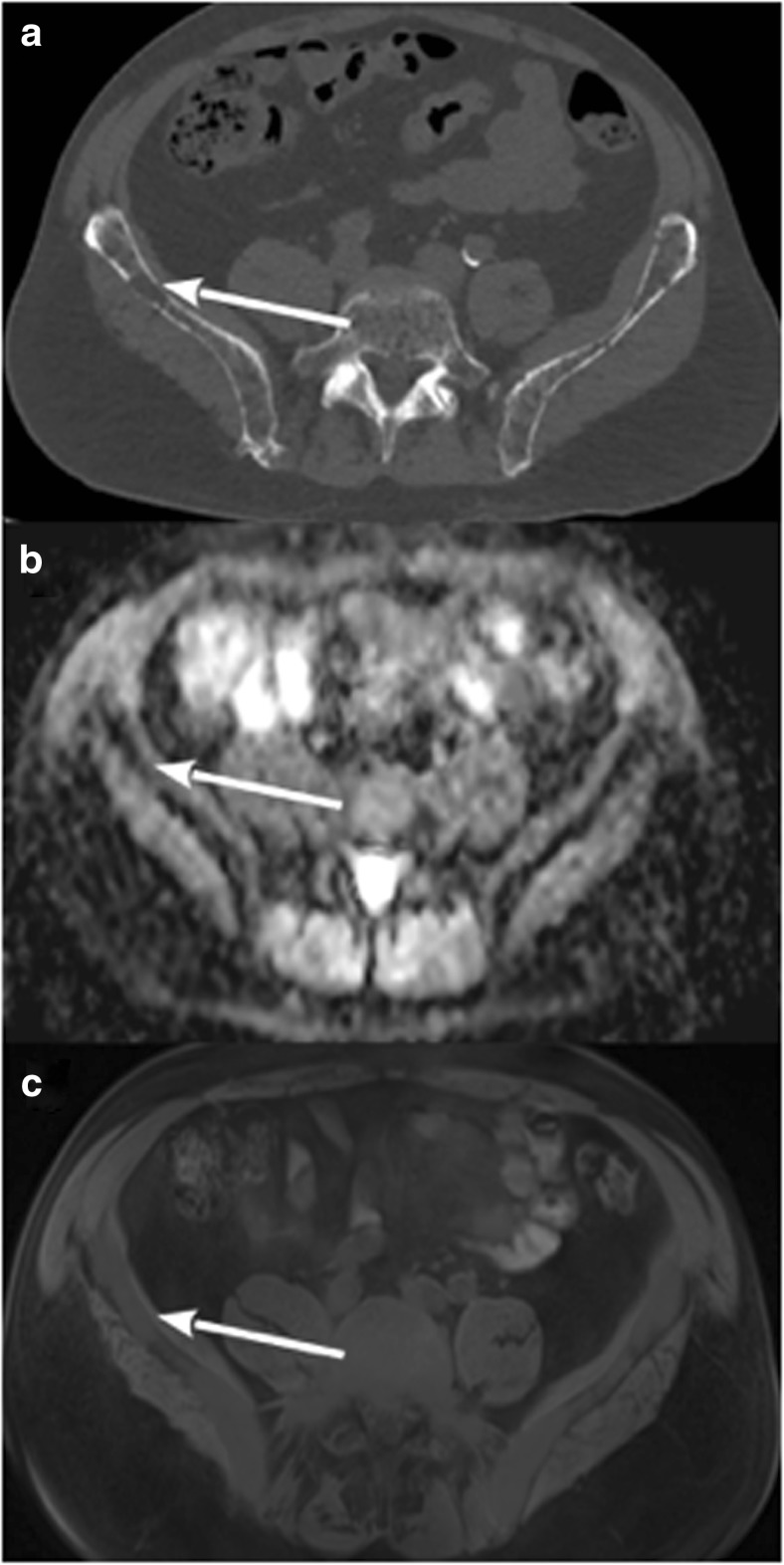
**a** WB-LDCT showing lytic bone lesion (white arrow) with negative densitometry of − 46 HU. **b** ADC map with ADC value of the lesion of 346 × 10^−6^ mm^2^/s. **c** Fused PET/MRI image showing normal FDG uptake of the area with SUVmax of 1.09

**Fig. 3:**
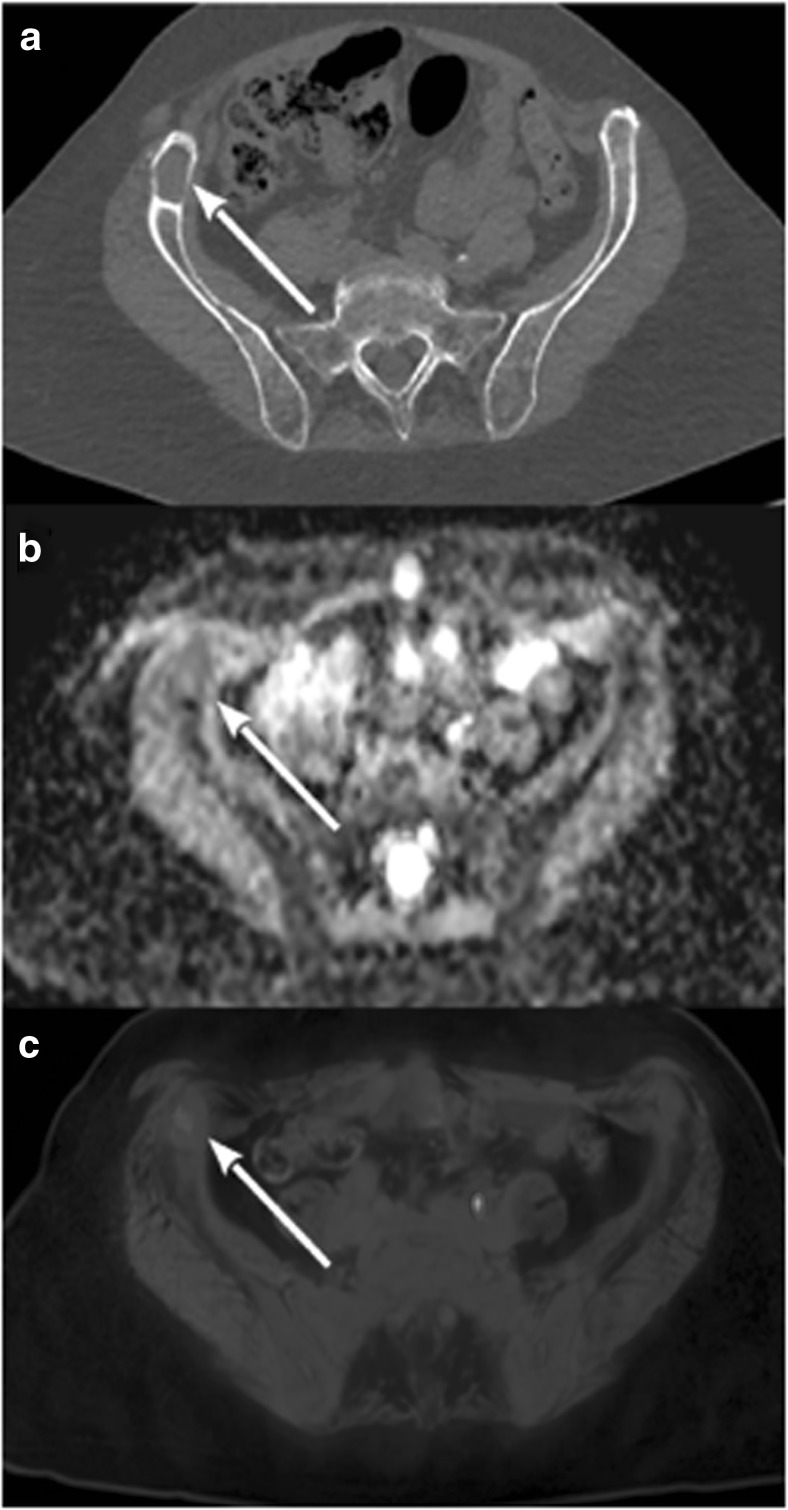
**a** WB-LDCT showing lytic bone lesion with positive densitometry of 53 HU. **b** ADC map with ADC value of the lesion of 876 × 10^−6^ mm^2^/s. **c** Fused PET/RMI image showing hypermetabolism of the lesion with SUVmax of 3.9

**Fig. 4:**
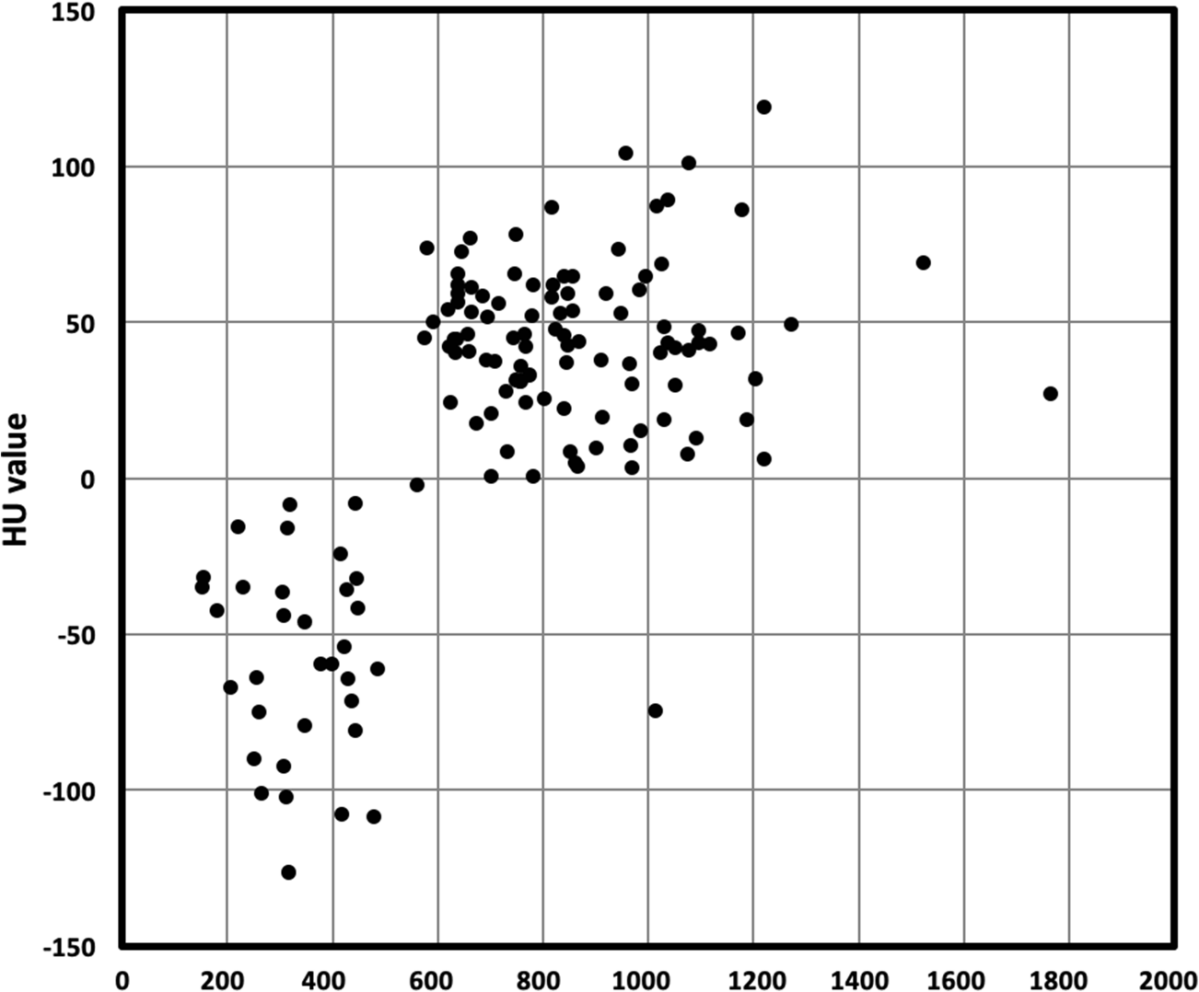
Scatter plot showing distribution of lytic bone lesion in relation to their densitometry (HU mean, *y*-axis) and ADC value (ADC mean, *x*-axis)

**Fig. 5:**
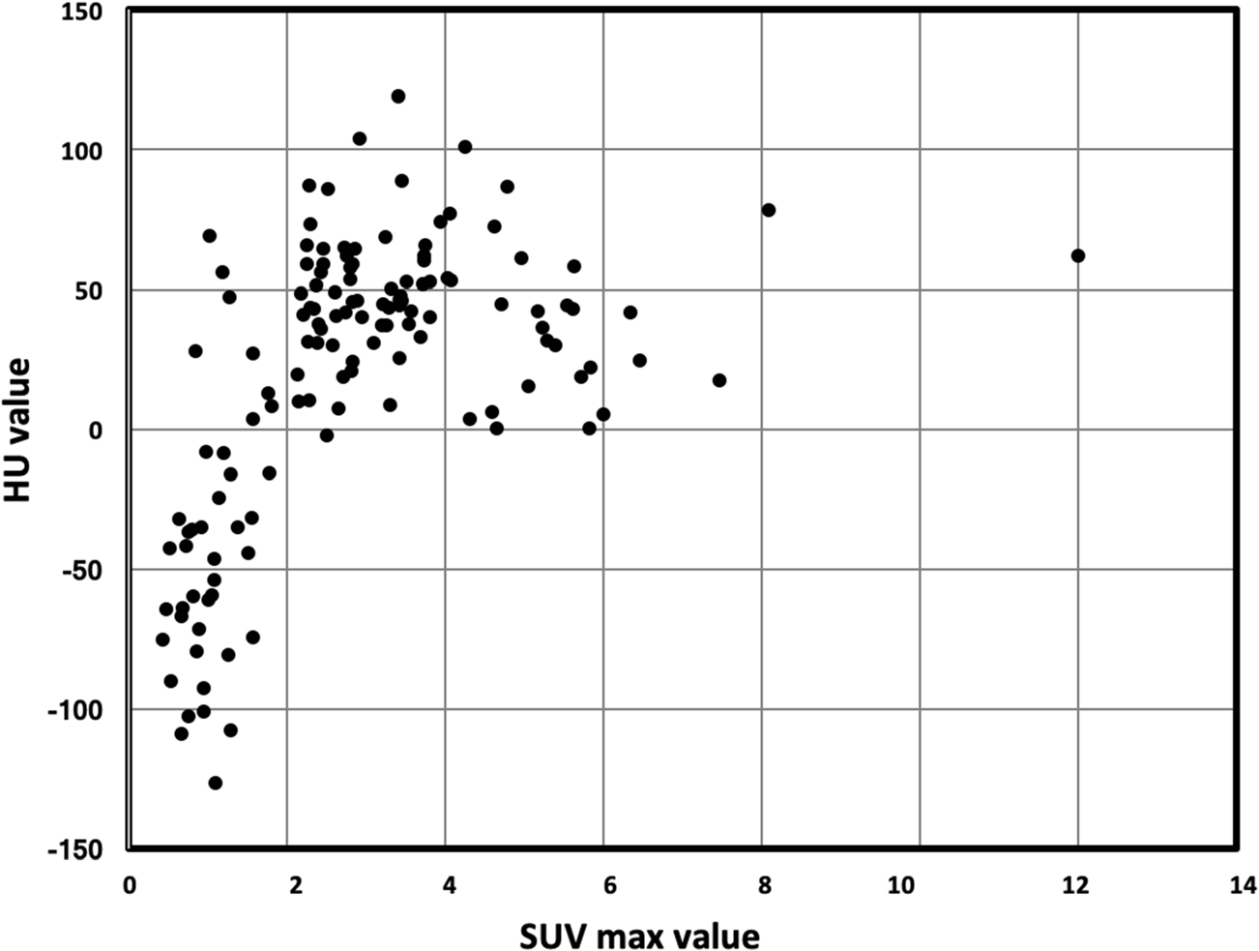
Scatter plot showing distribution of lytic bone lesion in relation to their densitometry (HU mean, *y*-axis) and SUVmax value (SUV mean, *x*-axis)

Patients with negative HU LBL showed low signal in STIR and DWI sequences, high signal in T1w images low ADC values (mean 363 × 10^−6^ mm^2^/s; SD ± 0.153) and low SUV max values (mean 1.03; SD ± 0.42) (Fig. 2 and supplementary Fig. 1; supplementary Fig. 2 reports another case with the same characteristics), while patients with positive HU LBL showed low T1w signal and high signal in STIR and DWI sequences, higher ADC values (mean 868 × 10^−6^ mm^2^/s; SD ± 0.208) and SUV max values (mean 3.54; SD ± 1.63) (Fig. 3). Statistical analysis based on linear regression with robust standard errors accounting for dependence between observations from the same patient confirmed that the mean ADC values and log-transformed SUV max values were strongly significantly different in the two groups (*z* = 10.7, *p* value < 0.001 for ADC; *z* = 10.793, *p* value < 0.001 for SUV max). Figures 4 and 5 display the relationships between densitometry and ADC and densitometry and SUV max, respectively.

### Histological analysis

**Fig. 6:**
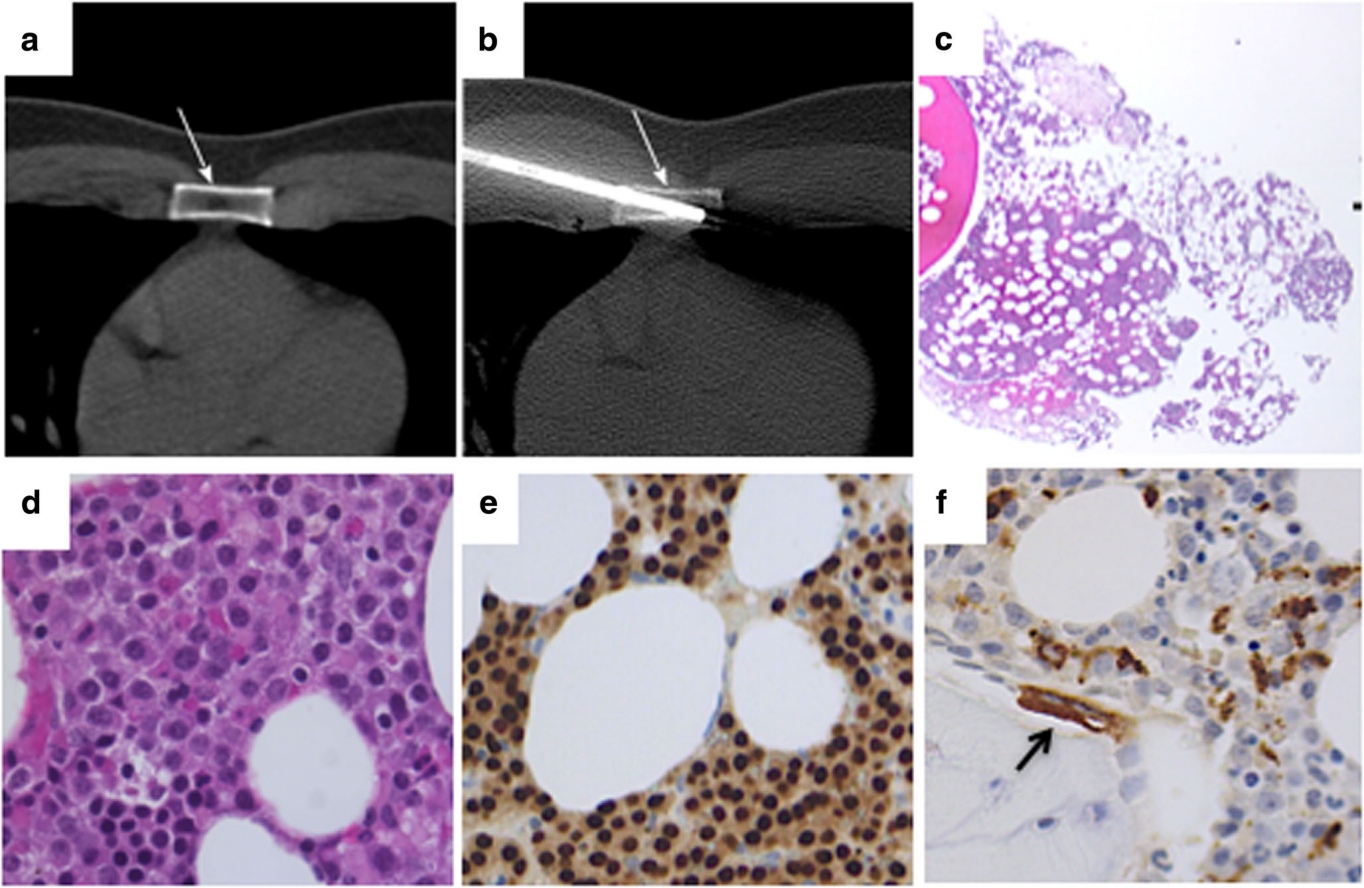
Histological evaluation of a representative biopsy on a negative HU lytic bone lesion. **a**, **b** Biopsy of lytic area performed under CT evaluation, at the site of the sternum. **c** Low magnification of bone marrow biopsy, showing the lack of bone trabeculae and the presence of plasma cells dispersed within adipocytes. **d** High magnification of H&E showing strong plasma cell infiltrate. **e** Same as before stained with MUM1. **f** The fatty cavity was surrounded by normal appearing bone marrow spaces with paratrabecular pluri-nucleated osteoclasts (arrow). (Histological images H&E and peroxidase stains, original magnification × 5, × 10, × 40)

Pelvis bone marrow biopsies were indicative for plasma cell disease in all patients, the PC percentage being more than 60% of total cells in 13 (72%) cases (Table 1). A CT-guided biopsy of representative osteolytic lesions with negative density was performed in four selected patients. In all cases, a plasma cell infiltrate with interstitial and confluent growth pattern, exceeding 10% of marrow cellularity, was demonstrated. The plasma cells were scattered in a loose stroma, mainly consisting of mature adipocytes with few (if any) intervening hematopoietic cells and/or marrow fibrosis. At the edges of the plasma cell infiltrate, occasional CD68-positive multinucleated osteoclasts were documented close to the bone trabeculae (Fig. 6).

## Discussion

According to HU densities of LBL, two different patterns of lytic lesions were herein demonstrated at WB-LDCT scan in MM patients, one with features of fat replacement of trabecular bone (HU < 0), the other characterized by pathological cell infiltration with tissue-like density (HU > 0). Only the infiltrative pattern was consistently captured by PET/MRI. Otherwise, WB-LDCT also detected unequivocal LBLs characterized by negative HU values, consistent with fat density, with negligible ^18^FDG uptake at PET and low ADC at DWI-MRI. Should only PET/MRI be taken into account in these cases, these types of lesions would have not been considered as a proof of symptomatic disease. We also demonstrated that both types of lytic lesions (i.e., those with HU < 0 and those with HU > 0) can be present at the same time in the same patient, highlighting that both conditions are consistent of symptomatic bone disease. As a matter of fact, the histological evaluation that typical negative density LBLs were characterized by infiltration of neoplastic plasma cells spreaded within adypocytes provided the formal proof of their pathological nature. Interestingly, we showed that, during follow-up, HU-negative LBLs can became HU positive with glucose avidity at PET and pathological ADC, suggesting a relationship between the two types of lesions, the fat pattern likely anticipating the infiltrative one.

Evidence of bone involvement can sometimes be demonstrated unexpectedly in otherwise fully asymptomatic MM patients, in some cases represented by a sole lytic lesion, making the decision to start therapy quite intriguing. This feature is not uncommon, since the presence of lytic lesions as unique CRAB criterium has been demonstrated in 40 out of 81 bone involvements as detected by WB-LDCT [16]. Furthermore, a unique 5 mm lytic lesion demonstrated by WB-LDCT is not regarded strength enough to decide that the patient must undergo systemic therapy, and in this case, just a short follow-up is suggested [17]. Although understandable, this approach suggests a certain degree of uncertainness, partly related to the recent availability of extremely sensitive techniques leading to unexplored levels of information, combining morphological with functional data. In line with this concern, neither in IMWG [9] nor the recently published ESMO guidelines [18] took into account the inner density of lesion detected by WB-LDCT. In addition, recently published recommendations from IMWG on interpretation of WB-LDCT suggest that the presence of fat-containing foci excludes the diagnosis of osteolysis, provided that the patient has not been previously treated [19]. Concordance between WB-LDCT and PET is reported to be higher than 85% [20] while concordance between WB-LDCT and MRI is roughly 70% [21, 22]. Considering that PET does not capture negative HU lytic lesions which can also be missed by DWI-MRI ADC evaluation with fat subtraction (MRI does not recognizes lytic lesions), the data reported in this study contribute to better characterize bone disease detected in MM patients, possibly accounting for some discrepancy between WB-LDCT, PET, and MRI. Bearing this in mind, our results suggest that the three techniques are not mutually exclusive, exploring nonoverlapping areas of bone involvement in MM patients. Provided these methodologies are available, the combination of each technique in the same MM patient could indeed offer the most precise and complete skeleton evaluation.

The pathogenetic mechanisms that lead to the development of myeloma bone disease are well known [23–26]. Since the first evidence in animal models that the RANKL/RANK/OPG system plays a critical role in the development of osteolytic bone disease in MM, data have been accumulated that bone resorption is associated with the presence of increased numbers of osteoclasts, whereas bone formation is reduced [27]. This uncoupling of resorption and formation, in association with an increased frequency of bone remodeling units, leads to rapid bone loss and the development of osteolytic bone lesions. Myeloma cells were found closely associated with CD68-positive multinucleated osteoclasts. Osteoclasts could be found on cancellous bone surfaces and were observed to line the corticoendosteal surface [23]. More recently, many chemokines and cytokines have been demonstrated to be involved into development of lytic lesions in MM patients [28–37], suggesting that humoral pathways are dominant in orchestrating the cross-talk between neoplastic plasma cells and osteoclasts. This hypothesis has been suggested by Dalla Palma et al. [38], who showed that MM patients with high burden of osteolytic lesions recognized by PET/CT are mostly characterized by high amounts of BM concentrations of CCL3 (MIP1a) and CCL20 (MIP3a), whereas BM DKK-1 levels were mostly associated with the presence of focal lesions on MRI in MM patients. In this quite heterogeneous landscape, the mechanisms according for the emergency of lytic lesion with HU < 0 values and fatty density is a matter of debate. The observation we provided that mean diameters of negative HU LBLs were significantly lower (11.09 mm ± SD 4.45 vs 17.36 mm ± SD 13.24) than that of infiltrative lytic lesions might suggest a particularly high cytokine/chemokine-secreting plasma cell clone. Alternatively, one might suggest that negative HU LBLs might represent an early stage, which anticipates a massive plasma cell infiltration. Data from one patient reported in this study (N°17) who was initially characterized by the presence of fatty lytic lesions only (HU − 47.7), then becoming within 10-month follow-up larger and featured by cell proliferation (HU + 29.5) are in line with the above interpretation (Fig. 1). Histological evaluation of this kind of HU < 0 lesion unequivocally demonstrated its pathological nature, characterized by a spread infiltration of neoplastic plasma cells, within a fatty background lacking bone structures, with accumulation of osteoclasts at residual bone surface (Fig. 6).

By fusing information of PET regarding prognostic value of number and intensity of lesions with that of MRI concerning different pattern of marrow involvement and ADC analysis, PET/MRI combines two techniques with a high morphological and functional potential in MM evaluation at the same time. Given the ability to recognize lesions on MRI that cannot be seen on PET (and vice versa), PET/MRI could be useful at any time point (initial staging and in residual disease detection) along evaluation of natural history of myeloma patients. Sachpekidis et al. demonstrated that PET/MRI shows equivalent performance as compared to PET/CT in terms of skeletal lesion evaluation in MM patients [39]. Recently published results, in a small series of MM patients with paired imaging data, suggest that patients with extensive disease on DWI-MRI may be reported as being disease-free on ^18^FDG-PET (“false-negative PET”) [40]. In a large series of cases, Rasche et al. found evidence of disease using DWI-MRI in areas without apparent involvement by PET in 11% of patients (26/227), with a concordance between the two techniques in 89% of cases [41]. These authors demonstrated that low expression of hexokinase-2 was associated with false negative PET. Our data confirm and extend the high concordance between ADC and PET activity in the definition of proliferative lesions. As a matter of fact, in terms of SUV and ADC, a significant concordance was found between PET and DWI-MRI for both positive and negative densitometry LBLs (91% and 94%, respectively), considering a cutoff value of SUV > 2 and ADC > 600 × 10^−6^ mm^2^/s as abnormal. Interestingly, when combined together as in PET/MRI, the contribution of MRI, namely ADC, in the detection of cellular lesions with HU > 0 resulted higher as compared to PET SUVmax (Figs. 3 and 4). This is likely due to the reduction (nearly 10%) of tracer uptake quantitation since the standard methods used for MR-based attenuation correction do not account for the presence of bone tissue in the attenuation map [39]. Taken together, with the limitations represented by the retrospective analysis, WB-LDCT detected lytic lesions that were missed not only by ^18^F-FDG but also by DWI-MRI, reinforcing the concept that for a proper skeletal survey in MM patients, data from the three techniques would be necessary, although several issues still remain open [39].

In conclusion, we report that the two types of lytic lesions recognized by WB-LDCT, although biologically different, should both be considered as evidence of bone disease and thus of symptomatic myeloma. These data are particularly relevant when negative HU lesions represent the only type of organ damage, since both ^18^F-FDG PET and DWI-MRI in these cases are unable to detect symptomatic disease.

## Electronic supplementary material

ESM 1 (DOC 7470 kb)
