# Supplementary material for: Whole-body low-dose CT recognizes two distinct patterns of lytic lesions in multiple myeloma patients with different disease metabolism at PET/MRI
Source: Ann Hematol. 2018 Dec 11;98(3):679–89. doi: 10.1007/s00277-018-3555-7 (PMC6373185; doi:10.1007/s00277-018-3555-7)
Supplement: Supplementary file 1 — (DOC 7470 kb) [file 277_2018_3555_MOESM1_ESM.doc]

**Supplementary**

**Figure 1**


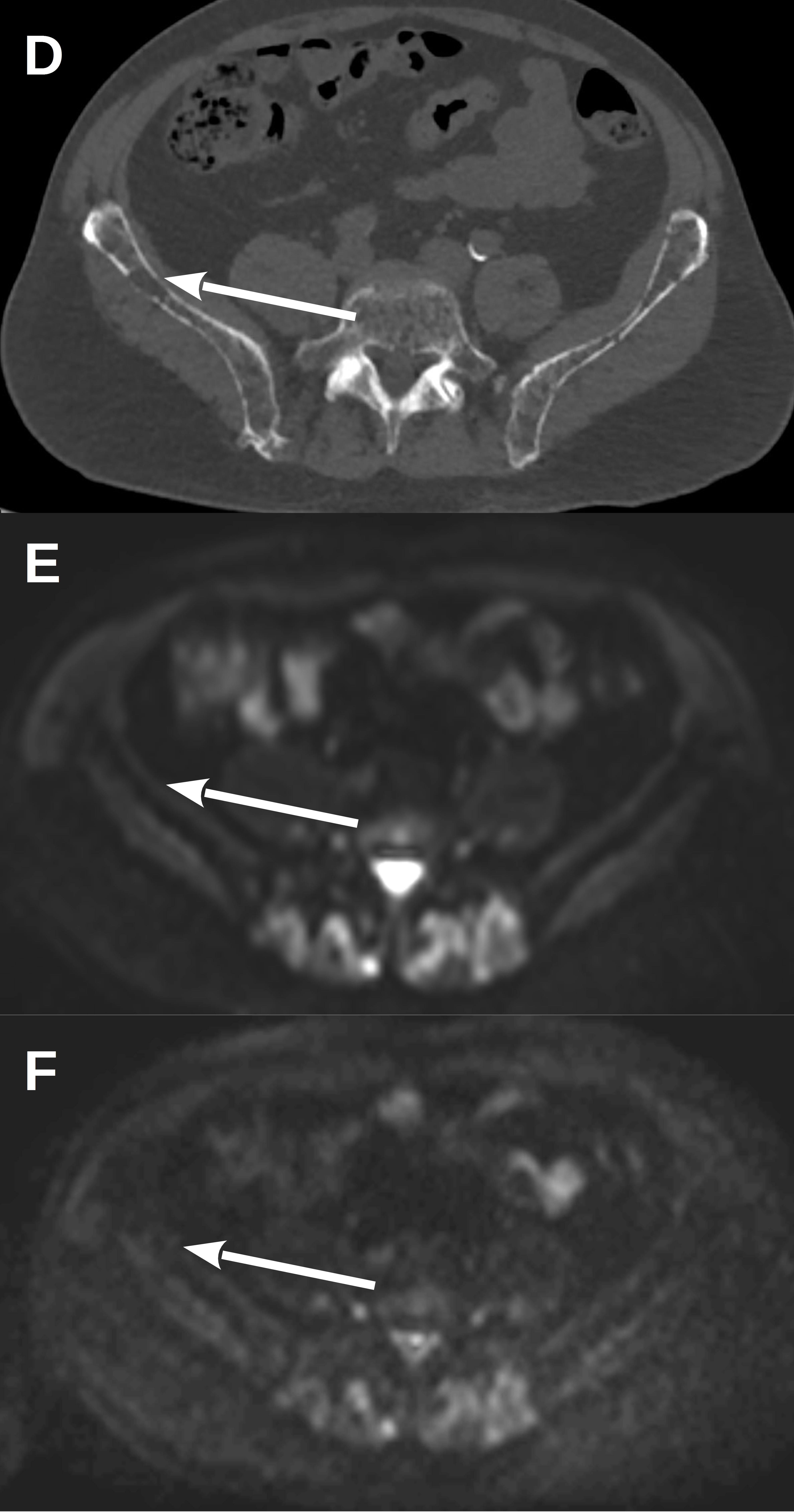

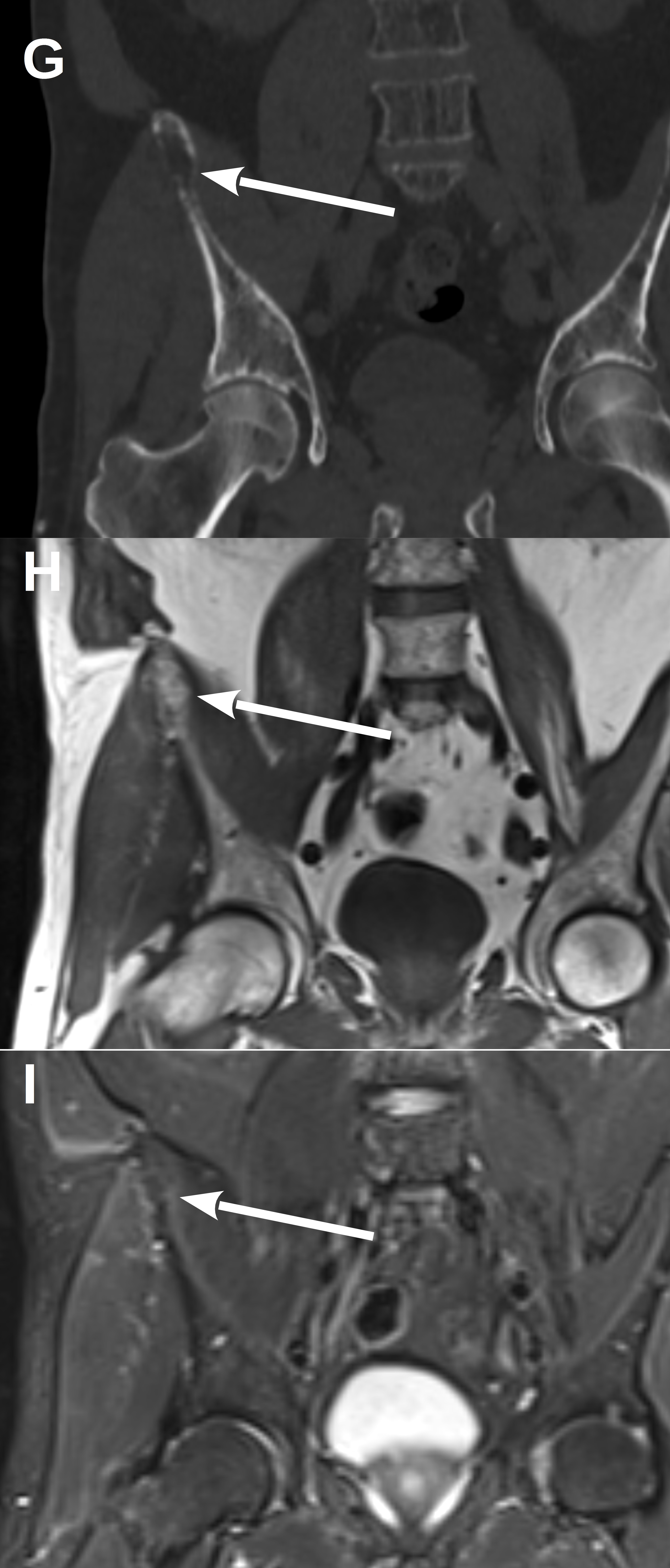


**Figure 2**


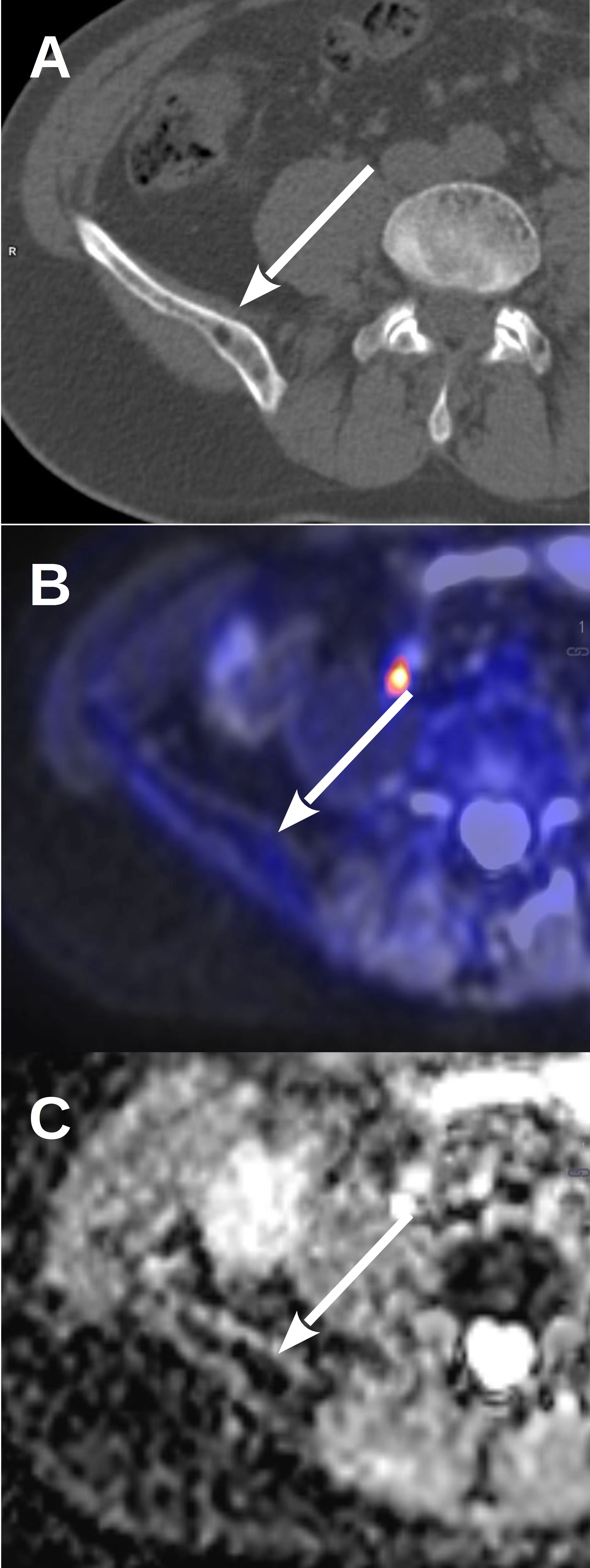

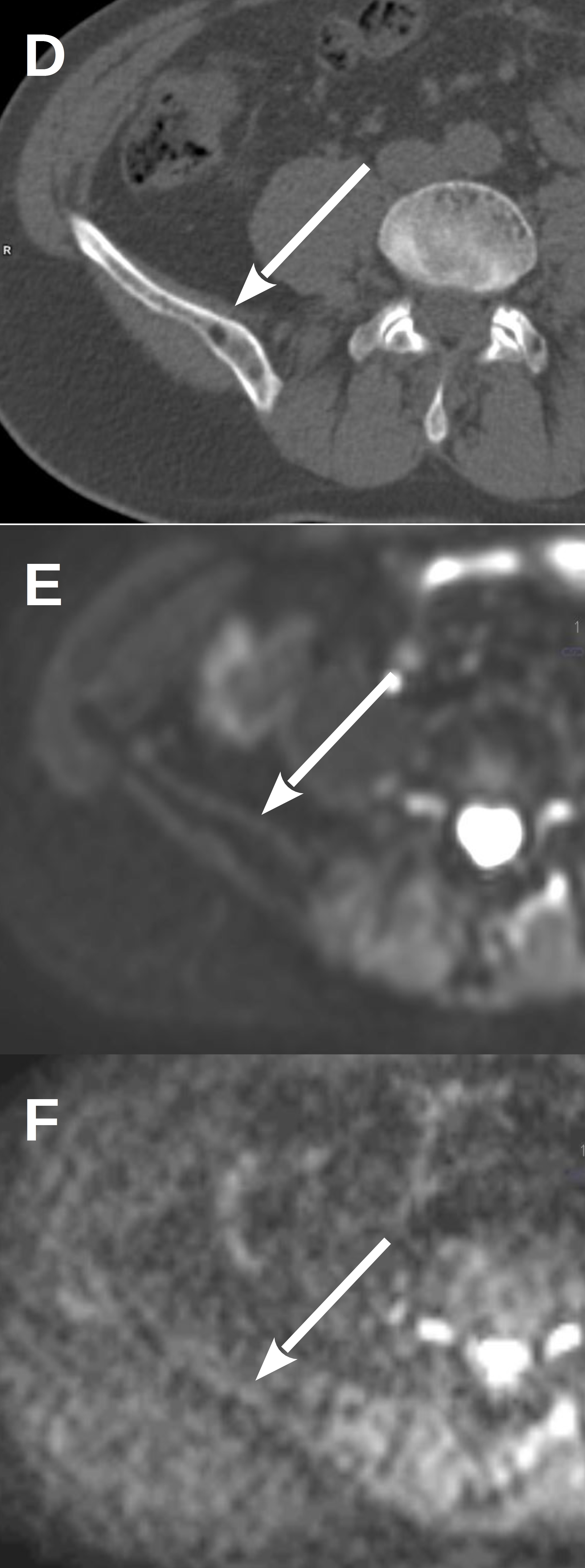

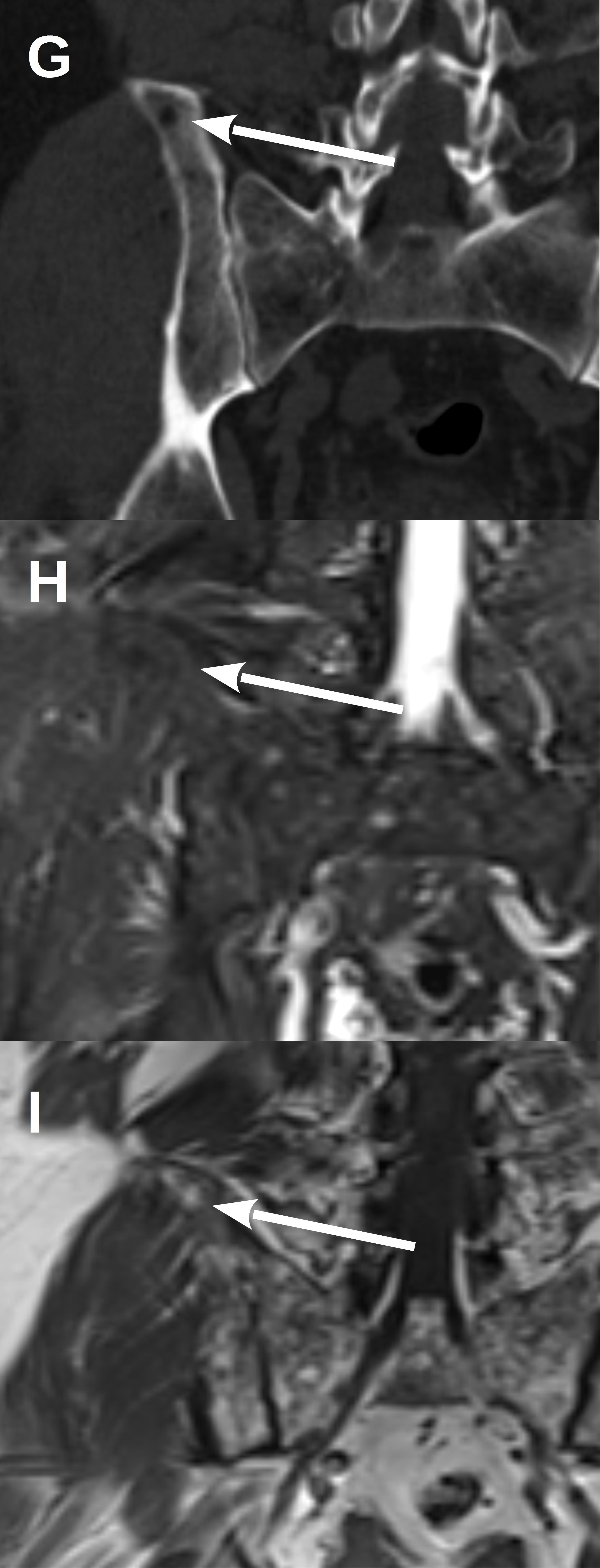


**Legends**

**Figure 1: D)** axial WB-LDCT image showing osteolytic lesion (white arrow) with negative densitometry; **E)** b50 DWI image showing no signal restriction in the same area; **F)** b1000 DWI image of the same lesion without signal restriction; **G)** coronal WB-LDCT scan of the negative densitometry lesion reported in figure 2A, main text; **H)** MRI T1-wighted coronal sequence of the same lesion showing no clear hypointense signal of the lesion; I) MRI T2-weighted fat-suppressed image showing no clear hyperintense signal of the lesion.

**Figure 2**: **A)** axial WB-LDCT image showing osteolytic lesion (white arrow) with negative densitometry (HU: -58) **B)** PET/MRI fused image without hypermetabolism of the lesion; **C)** ADC map showing no pathological ADC value of the same lesion; **D)** axial WB-LDCT image showing osteolytic lesion (white arrow) with negative densitometry (same as A); **E)** b50 DWI image showing no signal restriction in the same area; **F)** b1000 DWI image of the same lesion without signal restriction; **G)** coronal LDCT scan of the negative densitometry of the same lesion; **H)** MRI T1-wighted coronal sequence showing no clear hypointense signal of the lesion; **I)** MRI T2-weighted fat-suppressed image showing no clear hyperintense signal of the lesion.
